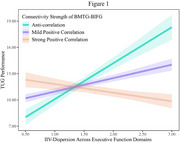# Brain connectivity moderating the association cognitive intraindividual variability and mobility in the cognitively frail older adults

**DOI:** 10.1002/alz70860_100036

**Published:** 2025-12-23

**Authors:** JINGYI WU, JINYU CHEN, JUNCEN WU, Chun Liang Hsu

**Affiliations:** ^1^ The Hong Kong Polytechnic University, Hong Kong, Hong Kong

## Abstract

**Background:**

Cognitive frailty is the concurrent presence of mild cognitive impairment (MCI) and physical frailty, causing one to be at greater risk for cognitive decline. Cognitive intraindividual variability (IIV) is a critical component of cognition involved in the maintenance of higher‐order processes under load. Greater IIV is hallmark of cognitive frailty and a marker for early signs of impaired cognition and mobility in older adults. However, the underlying neural mechanism of cognitive frailty‐related decline in IIV and mobility remains unexplored. This study aimed to clarify the association between brain function, IIV, and mobility in cognitively frail older individuals.

**Method:**

This cross‐sectional study included 17 cognitively frail older adults (CF) and 20 non‐cognitively frail older adults (non‐CF). Cognitive frailty was operationalized the presence of MCI (i.e., Montreal Cognitive Assessment score ≥ 18/30 and < 26/30) and physical frailty (i.e., Short Physical Performance Battery ≤ 9/12). All participants underwent clinical assessments including the Stroop Test, Trail Making Test, Timed‐Up and Go test (TUG), and resting‐state functional magnetic resonance imaging. Dispersion across executive tests was computed to ascertain IIV‐dispersion. Analysis of covariance was used to determine group differences in IIV‐dispersion, adjusting for the Functional Comorbidity Index. Moderation models were constructed to investigate the role of functional neural networks on the association between IIV‐dispersion and TUG performance.

**Results:**

Compared to non‐CF, CF exhibited greater IIV‐dispersion (*p* = 0.038), worse TUG performance (*p* <0.010), lower inter‐network connectivity in the DMN, FEN, and SMN (all *p* <0.050), as well as reduced intra‐network connectivity in the DMN and SMN (all *p* <0.050). Among CF, regional inter‐network connectivity between the DMN and FEN (i.e., bilateral middle temporal gyrus (BMTG) and bilateral inferior frontal gyrus (BIFG)) moderated the relationship between IIV‐dispersion and TUG performance (*R*‐sq=0.427, *p* = 0.001, Figure 1). Specifically, compared with individuals with lower BMTG‐BIFG connectivity (β=4.082, *p* <0.001), those with greater BMTG‐BIFG connectivity (β=1.561, *p* = 0.002) showed greater TUG performance under higher IIV‐dispersion load. These associations were not observed in non‐CF.

**Conclusion:**

Differences in intra‐ and inter‐network connectivity patterns in large‐scale functional neural networks between CF and non‐CF may underpin how IIV‐dispersion negatively impacts TUG performance in cognitively frail older individuals.